# Systematic Review of Genetic Polymorphisms Associated with Acute Pain Induced by Radiotherapy for Head and Neck Cancers

**DOI:** 10.1016/j.ctro.2023.100669

**Published:** 2023-08-19

**Authors:** Vivian Salama, Yimin Geng, Jillian Rigert, Clifton D. Fuller, Sanjay Shete, Amy C. Moreno

**Affiliations:** a Department of Radiation Oncology, The University of Texas MD Anderson Cancer Center, Houston, TX, USA; b Research Medical Library, The University of Texas MD Anderson Cancer Center, Houston, TX, USA; c Department of Biostatistics, The University of Texas MD Anderson Cancer Center, Houston, TX, USA

**Keywords:** Genetic variants, Polymorphisms, Radiation pain, Radiation mucositis, Radiation dermatitis, Head and neck cancers

## Abstract

**Background/objective:**

Pain is the most common acute symptom following radiation therapy (RT) for head and neck cancer (HNC). The multifactorial origin of RT-induced pain makes it highly challenging to manage. Multiple studies were conducted to identify genetic variants associated with cancer pain, however few of them focused on RT-induced acute pain. In this review, we summarize the potential mechanisms of acute pain after RT in HNC and identify genetic variants associated with RT-induced acute pain and relevant acute toxicities.

**Methods:**

A comprehensive search of Ovid Medline, EMBASE and Web of Science databases using terms including “Variants”, “Polymorphisms”, “Radiotherapy”, “Acute pain”, “Acute toxicity” published up to February 28, 2022, was performed by two reviewers. Review articles and citations were reviewed manually. The identified SNPs associated with RT-induced acute pain and toxicities were reported, and the molecular functions of the associated genes were described based on genetic annotation using The Human Gene Database; GeneCards.

**Results:**

A total of 386 articles were identified electronically and 8 more articles were included after manual search. 21 articles were finally included. 32 variants in 27 genes, of which 25% in inflammatory/immune response, 20% had function in DNA damage response and repair, 20% in cell death or cell cycle, were associated with RT-inflammatory pain and acute oral mucositis or dermatitis. 4 variants in 4 genes were associated with neuropathy and neuropathic pain. 5 variants in 4 genes were associated with RT-induced mixed types of post-RT-throat/neck pain.

**Conclusion:**

Different types of pain develop after RT in HNC, including inflammatory pain; neuropathic pain; nociceptive pain; and mixed oral pain. Genetic variants involved in DNA damage response and repair, cell death, inflammation and neuropathic pathways may affect pain presentation post-RT. These variants could be used for personalized pain management in HNC patients receiving RT.

## Highlights

• Pain is a complex symptom following radiation therapy for head and neck cancers.
• Acute pain control is highly challenging during radiotherapy in head and neck cancers.
• Inflammatory, nociceptive and neuropathic pain types are expressed after radiotherapy.
• Germline genetic variants are associated with radiation therapy related acute pain.
• Mechanisms of variants of radiation related pain are essential for pain management.

## Introduction

Head and neck cancers (HNC) are composed of a heterogeneous mix of malignant epithelial cancers that arise from the oral cavity, pharynx, or larynx. Oral cavity and oropharyngeal (OC/OPC) squamous cell carcinoma afflicted more than 54,000 patients in the U. S, in 2022 [1]. Management of head and neck cancer is a stage-dependent multimodality approach that entails surgery, chemotherapy, and radiotherapy (RT). RT is a cornerstone for the management of HNC patients. RT, especially after application of the novel RT techniques, has a favorable impact on the overall survival rates [2]. Despite improvement in survival rates, various significant acute and sometimes chronic toxicities are reported during and after RT. RT-attributable toxicities, including mucositis and dermatitis have significant negative impacts on patients’ outcome and quality of life (QOL) [3], [4], [5].

Oral/throat pain, the most common radiation-attributable acute toxicity during and after locoregional RT of HNC, is associated with increase in analgesics use [6]. About 68–86% of OC/OPC patients and about one-third of HNC patients present to the emergency department with uncontrolled pain [7], [8], [9], [10], [11]. The control of RT-induced acute pain is challenging because of the complex nature and multiple mechanisms of pain in these patients. Baseline pain and associated RT toxicities such as mucositis, dermatitis and neuropathy are considered the most common etiologies of RT-induced pain. In addition, pain due to surgery and/or chemotherapy are also observed in these patients [12], [13], [14]. Most clinicians will prescribe opioids to OC/OPC patients during cancer therapy, with 15–40% of patients dependent on opioids for several months post therapy [15], [16], [17]. While trying not to undertreat patients with severe pain, potential overuse of opioids may negatively impact the health status and the QOL of patients. Furthermore, for a subset of patients, the prolonged use of opioids increases the risk for drug abuse and addiction, which may negatively impact the QOL and increase the potential for overdose and death [18].

## The mechanism of radiotherapy-induced cell death and normal tissue toxicities

*Radiation therapy* is a group of ionizing energy beams that induce DNA damage in cells, cancer cells being most susceptible due to their characteristic of rapid cell division. Direct DNA damage is induced by ionizing radiation to DNA, inducing DNA breaks (single-strand breaks (SSBs), double strand breaks (DSBs)) and covalent crosslinking of the complementary DNA strands [19], [20]. Indirect DNA damage occurs through RT-induced generation of free radicals. For instance, reactive oxygen species (ROS) are produced by the interaction of ionizing radiation with the water molecules [21]. After RT, the impairment of DNA repair mechanisms in cancer cells in addition to the accumulation of intracellular ROS induces cell injury and apoptosis, necrosis, and cellular senescence [22], [23], [24], [25]. However, normal neighboring healthy tissues will also be exposed to ionizing radiation, inducing normal cell injury and potential cell death despite the lower doses of radiation exposure. Normal tissue damage induces toxicities such as oral mucositis, dermatitis, and neuropathy leading to aggravation of RT-induced acute pain. The severity of RT-induced toxicities and pain is dependent on the total dose, fractions and the duration of radiation delivered to normal tissue in addition to the variation in tissue tolerance to radiation [26], [27].

## Types and potential mechanisms of radiotherapy induced acute pain in HNC patients

**Fig. 1:**
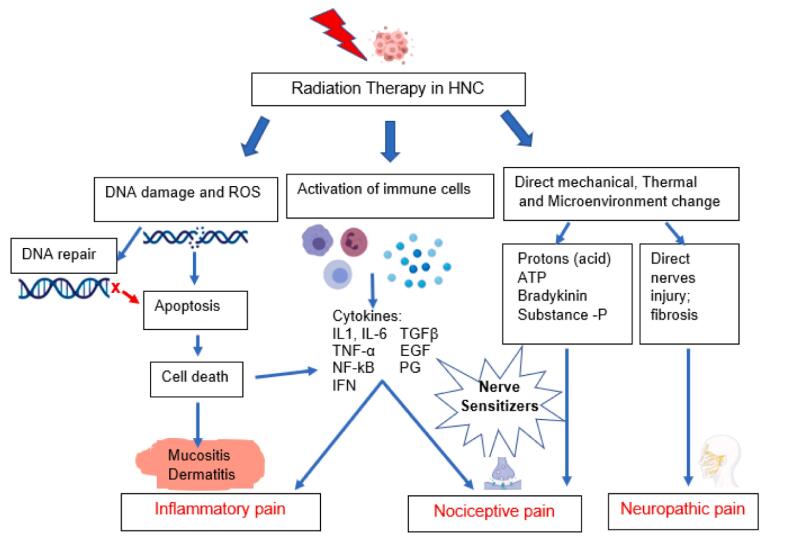
**Types and potential mechanisms of radiotherapy induced acute pain. 1-** Radiotherapy in head and neck cancer induces DNA damage of cancer cells and normal neighboring cells and the release of reactive oxygen species (ROS). DNA damage induces activation of DNA repair mechanisms which is impaired in cancer cells. Impairment of DNA damage repair induces activation of cell death through stimulation of apoptosis processes. Normal tissue death induces mucositis and dermatitis causing **inflammatory pain**. **2-** Radiotherapy induces activation of immune responses and inflammatory cells which release inflammatory mediators such as cytokines and chemokines inducing inflammatory pain. Furthermore, these inflammatory mediators act as noxious stimuli, stimulating nociceptors to induce **nociceptive pain**. **3-** Radiotherapy induces direct mechanical and thermal damage in addition to changes in the microenvironment creating an acidic pH, increasing the adenosine triphosphate (ATP), and stimulating release of bradykinin and substance P which act as nociceptors stimuli inducing nociceptive pain. Radiotherapy induces direct nerve injury and inflammation of peripheral nervous system, and fibrosis in the connective tissue can also induce **neuropathic pain**.

Adequate management of acute pain in NHC patients during RT requires good understanding of the different types and mechanisms of RT-induced pain. Three main types of RT-induced acute pain have been identified in HNC patients receiving RT [28], [29], [30] Fig. 1:

1. Inflammatory pain
2. Nociceptive pain
3. Neuropathic pain

### Inflammatory pain

Inflammatory pain, the most common type of RT-induced acute pain in HNC, is caused by activation of inflammatory and immune cells in response to tissue injury and infections induced by RT [21], [31]. Inflammatory and immune cells in addition to RT-induced damaged cells release inflammatory mediators such as pro-inflammatory cytokines: interleukin *(IL)-1β*, *IL-6*, *NF-kB*, prostaglandins *(PG)*, and tumor necrosis factor-α (*TNF-α*) [21], [32], [33]. These cytokines promote recruitment of immune and inflammatory cells, inducing more damage of tissues [34]. Oral/pharyngeal mucositis and neck dermatitis are the most common inflammatory reactions induced by RT [21], [33], [35], [36]. Different signaling pathways involved in RT-induced mucositis and dermatitis have been identified, and they may contribute to RT-induced inflammatory pain, such as: nuclear factor-κB (*NF-κB*) signaling which upregulates other pathways such as COX2 pathway and downstream tyrosine kinase receptor pathways (e.g., *PI3K/AKT* signaling and *MAPK* signaling). Other signaling pathways such as DNA damage checkpoints, cell cycle pathways, *WNT/B-catenin* and integrin signaling, *VEGF* signaling, glutamate receptor signaling and *IL-6* signaling, have also been identified in correlation with RT-induced mucositis [36], [37].

### Nociceptive pain

Nociceptive pain is caused by direct damage to non-neural tissue, often from an external injury (i.e., RT-associated) which stimulates nociceptive receptors [30]. Noxious stimuli are released from either damaged cancer cells, normal cells or from inflammatory/immune cells recruited after RT [38]. Noxious stimuli activate the peripheral sensory neurons through stimulation of afferent sensory neurons which transmit the action potential to neuronal bodies where calcium influx occurs, leading to the release of neurotransmitters (e.g.: substance-P, glutamate, γ-aminobutyric acid (*GABA*), adenosine triphosphate (*ATP*), glycine, dopamine, norepinephrine, nitric oxide, and serotonin) which bind to the post-synaptic membrane receptors. The signal then reaches the second order neuron and is transmitted to the somatosensory cortex of the brain where the pain is perceived [39], [40], [41], [42], [43]. The trigeminal nerves (cranial nerve (CN) V)) and the facial nerves (CN VII) play important roles in pain perception in the head and neck [44]. RT-damaged cells and infiltrating immune cells release multiple noxious stimuli. Cytokines and inflammatory mediators released from infiltrating immune cells such as *IL-1*, *TNF-α* and *IL-6* induce nociceptor sensitization. In addition, the acidic pH of the tumor microenvironment and extracellular ATP act as noxious stimuli to nociceptors at the cancer site [45].

### Neuropathic pain

Neuropathic pain is caused by damage to nerves or nervous system [46]. RT directly injures the somatosensory nervous system promoting pain signal transmission and peripheral neuropathic pain. RT induces DNA damage which induces apoptosis. Apoptosis is induced through p53 activation which activates the cascade to execute cell death [47], [48]. Endothelial cell death reduces blood flow to peripheral nerves, including damage and eventual neuronal fibrosis. Furthermore, ionizing radiation and ROS released after RT, cause neuronal cell stress and direct nerve damage. In RT of HNC, the brachial plexus are the neural tissues at high risk of damage. Overexpression of *p53* induces apoptosis of neurons after radiation exposure [49]. Furthermore, RT induces inflammation and infiltration by immune cells, resulting in immune-mediated peripheral neuropathy. Chronic inflammation in the nervous system microenvironment promotes neuron loss with fibrosis, resulting in chronic neuropathic pain [50].

## Genetic variants associated with radiotherapy induced acute pain

Advances in molecular and genetic technologies have motivated researchers and scientists toward exploring the human genome and analyzing genetic variations and their correlations with different diseases and treatment outcomes.

RT-induced acute pain presents a significant morbidity burden on HNC patients receiving RT and drastically reduces patients’ quality of life. In an effort to better predict and optimize pain management approaches, various cellular and molecular approaches have been widely explored recently to identify the genomic biomarkers for patients vulnerable to develop RT-induced pain, and examine the influence of these genetic variants on pain modulation and analgesic response [51], [52]. The advanced technologies in human genome sequencing allowed deep understanding of the genetic variations and mutations related to cancer treatment associated pain. Single nucleotide polymorphisms (SNPs) are the most common DNA sequence variations. These genetic polymorphisms are stable markers and easily and reliably assayed to explore the extent to which genetic variation might prove useful in identifying patients with cancer at high-risk of pain development and their response to pain therapies [53]. Likewise, these candidate SNPs could be used in building robust predictive models for pre-treatment prediction of acute as well as chronic toxicities for personalized management and precision medicine [54].

Given the multifactorial and complex etiology of RT-induced pain in HNC patients, the challenges in managing RT-associated pain, and the adverse impact of pain on patients QOL, the aim of this literature review is to identify predictive genetic variants and pathways associated with RT-induced pain and related phenotypes in the HNC population. The review will focus on oral mucositis (OM) and dermatitis-related pain which represent common acute, often painful toxicities during HNC radiation treatment. This synthesis of the current literature will provide the basis to develop predictive models of RT-induced acute pain to assist with personalized analgesic therapy.

## Materials and methods

### Search strategies

We conducted a systematic search of databases including Ovid MEDLINE, Ovid Embase, and Clarivate Analytics Web of Science, for publications in English language from the inception of databases to February 28, 2022. The following concepts were searched using subject headings and free text keywords as needed, “radiotherapy”, “radiation therapy”, “pain”, “neuropathy”, “analgesics”, “acute toxicity”, “mucositis”, “dermatitis”, “single nucleotide polymorphism”, “genetic variation”, “genetic variability”, and “genetic predisposition”. The terms were combined using AND/OR Boolean Operators. Animal studies, in vitro studies, and conference abstracts were removed from the search result. Manual searches of journals, publisher databases and reference lists of journal articles were also conducted to supplement the electronic database search. The complete electronic database search strategies were detailed in Supplementary Tables S1–S3.

### Inclusion criteria

The included studies met all of the following inclusion criteria: 1) articles published in English, 2) human studies in head and neck cancers, 3) genetic polymorphisms reported to be significantly associated with RT-induced pain in different phenotypes of pain (Inflammatory [oral mucositis, dermatitis], neuropathy, nociceptive pain, or mixed oral/throat pain).

### Exclusion criteria

**Fig. 2:**
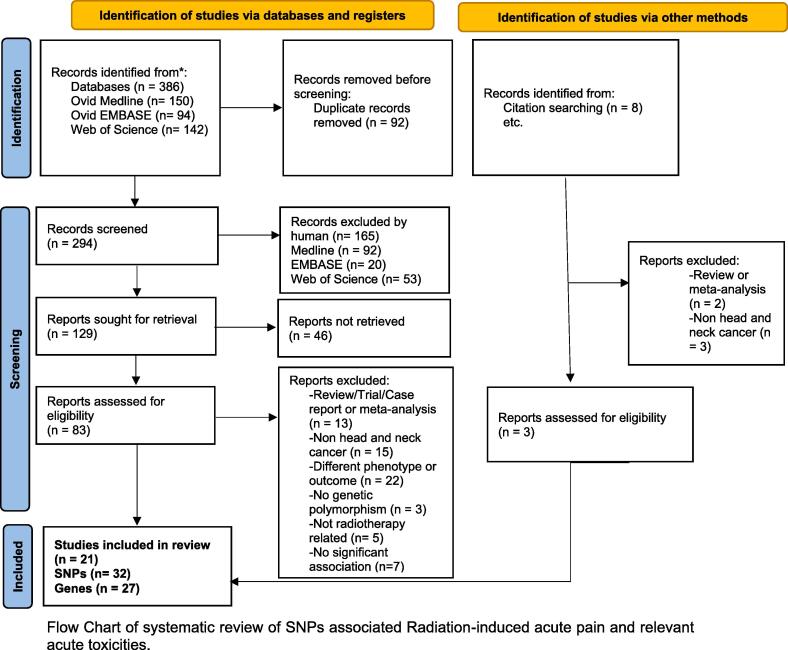
Flow Chart

Articles were excluded if they met any of the following criteria: 1) non-genetic article or no genetic association study, 2) not radiotherapy induced pain or toxicity, 3) *meta*-analysis, review article or clinical trial, 4) articles written in languages other than English, 5) other cancer types (i.e., non HNC) 6) non-human study, or not blood or buccal DNA or 7) Other unrelated phenotypes. Flow Chart Fig. 2

.

### Functional annotation

Manual functional annotation of the identified genes was done using GeneCards database. The function of each gene was assigned according to the most common functional pathway annotated in the GeneCards and the relevant studies published online. [55], [56] We divided the collected variants and genes into three groups: 1) variants associated with RT-induced inflammatory pain including; RT-induced acute toxicities; acute mucositis and acute dermatitis (or acute skin reaction), 2) variants associated with radiotherapy induced neuropathic pain, and 3) variants associated with other mixed types of RT-induced acute pain (including post RT-pain, post-RT throat/mouth/neck pain).

## Results

### Variants associated with radiotherapy induced inflammatory pain

Acute oral/pharyngeal mucositis and acute skin reactions are the most common acute reactions reported following RT. Nineteen articles were identified in our review detected SNPs significantly associated with acute mucositis or dermatitis post-RT. Twenty-three variants in 20 genes were associated with RT-induced inflammatory pain and acute reactions including mucositis or dermatitis. (Table 1). 5 (25%) genes are involved in inflammatory pathways and immune systems, 4 (20%) genes are involved in DNA damage and DNA repair pathways, 4 (20%) genes regulate cell death or cell cycle pathways, 2 (10%) genes are involved in metabolisms and microenvironment functional pathways, 5 (25%) genes are functionally involved in other functional pathways (Table 2).

**Table 1:** Summary of the literature search of variants associated with RT-induced acute inflammatory pain.

SNP	Gene	Function/Pathway	Phenotype	Sample/cancer	Number of patients	Author/year	OR	95% CI	*P value*
rs28419191	*STING1*	Inflammation/immune system	Mucositis	Blood DNA/HNC	1780	Schack et al./2022. [57]	2.27	1.70–3.05	4.4E-08
rs10875554	*STING1*	Inflammation/immune system	Mucositis	Blood DNA/HNC	1780	Schack et al./2022. [57]	2.25	1.68–3.01	5.1E-08
rs1131769	*DNAJC18*	Chaperone molecules protecting other cellular proteins	Mucositis	Blood DNA/HNC	1780	Schack et al./2022. [57]	2.21	1.66–2.95	7.6E-08
rs1265081	*CCHCR1*	keratinocyte proliferation or differentiation/EGFR	Mucositis	Blood DNA/HNC	500	Li et al./2021. [58]	1.41	1.08–1.86	0.012
rs3135001	*HLA-DQB1*	MHC class II, immune system	Mucositis	Blood DNA/HNC	500	Li et al./2021. [58]	0.53	0.35–0.79	0.002
rs117157809	*TNKS*	Telomere capping and maintenance and telomerase activity	Mucositis	Blood DNA/NPC	1467	Yang et al./2020. [59]	3.72	2.10–6.57	6.33E − 6
rs1799964	*TNF-α*	Proinflammatory cytokines	Mucositis	Blood DNA/HNC	62	Mlak et al./2020. [60]	6.61	2.14–20.40	0.001
rs1799782	*XRCC1*	DNA repair	Oral mucositis	Blood DNA/laryngeal cancer	150	Raturi et al./2020. [61]	6.9	1.4–34.7	0.01
rs1799782	*XRCC1*	DNA repair	Dermatitis	Blood DNA/laryngeal cancer	150	Raturi et al./2020. [61]	2.0	1.0–3.6	0.02
rs10898880	*ATG16L2*	Autophagy	Mucositis	Blood DNA/NPC	468	Yang et al./2019. [62]	1.56	1.19–2.04	0.001
rs10514231	*ATG10*	Autophagy	Mucositis	Blood DNA/NPC	468	Yang et al./2019. [62]	1.95	1.31–2.9	0.001
rs25487	*XRCC1*	DNA repair	Mucositis	Blood DNA/OPC	179	Gupta et al./2019. [63]	2.629	1.136–6.087	0.024
rs4149570	*TNFRSF1A*	TNF-α Proinflammatory	Mucositis	Blood DNA/HNC	58	Brzozowska et al./2018. [64]	5.625	1.584–19.975	0.008
rs1629816	*GHRL*	Modifier of proinflammatory cytokine activity	Mucositis	Blood DNA/HNC	65	Brzozowska et al./2018. [65]	5.92	1.58–22.22	0.008
rs4855883	*APEH*	Proteasome degradation	Mucositis	Blood DNA/HNC	62	Brzozowska et al./2018. [66]	0.23	0.07–0.77	0.0166
rs9344	*CCND1*	Cell cycle.	Mucositis	Blood DNA/NPC	154	Guo et al./2017. [67]	0.08	0.01–0.66	0.019
		NF-κB signaling							
rs1800541	*EDN1*	Angiogenesis-related genes	Mucositis	Blood DNA/NPC	180	Ma et al./2017. [68]	2.020	1.039–3.928	0.038
rs11081899	*ZNF24*	DNA replication, and DNA damage response, NF-κB activation, tumor progression and angiogenesis	Mucositis	Blood DNA/NPC	33	Le et al./2017. [69]	14.631	2.61–105.46	1.2E10 − 4
rs375557	*GSK3β*	Wnt/β-catenin pathway	Mucositis and dermatitis	Blood DNA/NPC	188	Yu et al./2016. [70]	2.34	1.05–5.21	0.033
rs454886	*APC*	Wnt/β-catenin pathway	Mucositis and dermatitis	Blood DNA/NPC	188	Yu et al./2016. [70]	1.57	1.01–2.43	0.045
rs1805794	*NBN*	DNA damage response, DNA repair	Mucositis	Blood DNA/HNC	183	Venkatesh et al./2014. [71]	3.750	1.201–11.70	0.023
rs132788	*KU70*	DNA repair	Mucositis	Blood DNA/NPC	120	Ren et al./2014. [72]	3.041	1.257–7.360	0.014
rs25487	*XRCC1*	DNA repair	Mucositis and dermatitis	Blood DNA/NPC	114	Li et al./2013. [73]	2.65	1.04–6.73	0.037
rs25487	*XRCC1*	DNA repair	Mucositis	Blood DNA/HNC	101	Pratesi et al./2011. [74]	4.02	1.16–13.90	0.025
rs2067079	*GAS5*	Glucocorticoid receptors. Apoptosis and cell cycle.	Oral mucositis	Blood LncRNA/NPC	238	Guo et al./2017. [75]	3.031	1.014–9.055	0.047
rs6790	*GAS5*	Glucocorticoid receptors. Apoptosis and cell cycle.	Dermatitis	Blood LncRNA/NPC	238	Guo et al./2017. [75]	0.026	0.001–0.464	0.013

OR: Odds ratio, CI: Confidence Interval.

**Table 2:** Pathways and biological functions of genes associated with RT-induced acute inflammatory pain.

Pathway	Genes	Number (n)	Percent (%)
Inflammatory/Immune system	*STING1, HLA-DQB1, TNF-α, TNFRSF1A, GHRL*	5	25 %
DNA damage/DNA repair	*XRCC1, ZNF24, NBN, Ku70*	4	20 %
Cell death/Cell cycle	*ATG16L2, ATG10, CCND1, GAS5*	4	20 %
Metabolism/Microenvironment	*APEH, EDN1*	2	10 %
Others	*DNAJC18, CCHCR1, TNKS, GSK3β, APC*	5	25 %

Different grading scales and cut points for toxicity identification were used in the identified studies as well as different covariates and clinical characteristics were considered in data analysis. (Table 3).

**Table 3:** Grading scale, cut points and covariates in studies of RT-induced inflammatory pain.

Article	Grading Scale	Cut points	Covariates/clinical characteristics
Schack et al./2022. [57]	STAT scores; 1: erythema, 2: patchy, 3: confluent and 4: ulceration	Binary cut points: “moderate/severe”: 0–2 vs 3–4	Sex, age, total RT dose, chemotherapy, irradiated volume protocol, and a surrogate
		Binary cut point ‘severe’’: 0–3 vs 4	
Li et al./2021. [58]	RTOG/EORTC	Two groups: (grade 0–2) group and (grade 3–4) group	–
Yang et al./2020. [59]	RTOG/EORTC	Two groups: severe OM (grade ≥ 3) and mild OM (grade ≤ 2)	Treatment scheme, radiation technology and the first five eigenvectors of principal component
Mlak et al./2020. [60]	RTOG/EORTC	–	Gender, age, Tobacco smoking, Alcohol consumption, histopathological diagnosis.
			TNM stage, TNF-α plasma concentration
Raturi et al./2020. [61]	CTCAE v4.03	Binary outcome as yes or no according to the grade.	Sex, age, tumor subsite and stage, Tobacco chewing or smoking and alcohol consumption
Yang et al./2019. [62]	RTOG/EORTC	Two groups: “non‐sensitive or mildly radiosensitive (grade 0–2) and “highly radiosensitive” (grade 3–4)	Gender, age, BMI, smoking, drinking, family history of cancer, EBV‐DNA, and TNM stage chemotherapy
Gupta et al./2019. [63]	CTCAE v4.0	–	Karnofsky Performance Status, smoking and tobacco chewing
Brzozowska et al. [64], [65], [66]]	RTOG/EORTC	–	demographic-clinical factors
Guo et al./2017. [67]	RTOG/EORTC	Two groups: “non-sensitive or mildly radiosensitive” (grade 0–2), and “highly radiosensitive” (grade 3–4).	Gender, age, gender, smoking, drinking, BMI carcinoma stage, and chemotherapy
Ma et al./2017. [68]	RTOG/EORTC	Two groups: “radiosensitive toxic reaction” (grade ≥ 3), radiation insensitive mild toxicity (grade < 3)	Gender, age, drinking, smoking, BMI, family history, TNM stage and clinical stage
Le et al./2017. [69]	CTCAE v3.0	Two groups: (CTC 0–2) group and (CTC 3 + ) group.	–
Yu et al./2016. [70]	RTOG/EORTC	Two groups: “non-sensitive or mildly radiosensitive” group (grade 0–2) and a “highly radiosensitive” group (grade 3–4)	Sex, age, BMI, TNM stage, smoking and drinking, family history, and chemotherapy
Venkatesh et al./2014. [71]	RTOG	Two groups: Grade ≤ 2 OM and Grade > 2 OM and	–
Ren et al./2014. [72]	CTCAE v3.0	Two groups: (CTC 0–2 toxicity grades) group, and (CTC 3 + ) group.	Demographics and clinical features.
Li et al./2013. [73]	CTCAE v3.0	The highest grade of toxicity was chosen as the reference value	Gender, age, smoking, drinking, BMI, tumor stage, RT technique, and radiation dose to observed tissue volumes
Pratesi et al./2011. [74]	CTCAE	Development of acute toxicity of Grade 2 was considered as increased sensitivity for acute RT effects	Biologically effective radiation dose (BED)
Guo et al./2017. [75]	CTCAE v3.0	Two groups: “severe toxicity” (grade 3–4), and “mild toxicity” (grade 1–2)	Clinical covariates: gender, age, BMI, Smoking, drinking, clinical stage, CCRT regimen, irradiation dose, myelosuppression, anemia, and thrombocytopenia

RTOG/EORTC: Radiation Therapy Oncology Group or European Organization for Research and Efficacy of Cancer, CTCAE: The Common toxicity criteria for adverse event, TNM: Tumor, nodal, metastasis staging, BMI: Body mass Index, EBV: Epstein–Barr virus. CCRT: Concurrent chemo-radiotherapy.

### Variants associated with radiotherapy induced neuropathic pain

Neuropathic pain is one of the common types of pain developed after receiving RT in HNC. Few studies focused on identification of SNPs associated with radiotherapy-induced neuropathic pain. Reyes-Gibby et al. study was the only literature published to identify SNPs associated with neuropathy and neuropathic pain in HNC. In this study, the authors used the International Classification of Diseases, ninth (ICD-9) and tenth (ICD-10) revisions, for the outcome toxicity identification (i.e., neuropathy/neuropathic pain), they used a binary outcome of neuropathy either yes or no. Age, sex and information of clusters were used as covariates in the statistical analysis. 4 variants in 4 genes were identified, associated with radiotherapy induced neuropathic pain. (Table 4).

**Table 4:** Summary of literature search of variants associated with RT-induced acute neuropathic pain.

SNP	Gene	Function/Pathway	Phenotype	Sample/cancer	Number of patients	Author/year	OR	95 %CI	*P value*
rs10950641	*SNX8*	Endocytosis, endosomal sorting and signaling. Glutamatergic-receptor-dependent neural plasticity	Neuropathic pain	DNA/HNC	1,043	Reyes-Gibby et al./2018 [76]	2.88	2.19––3.79	3.39E-14
rs6796803	*KNG1*	kinins act as mediators of pain and inflammation	Neuropathic pain	DNA/HNC	1,043	Reyes-Gibby et al/2018 [76]	0.51	0.41––0.64	6.42E-09
rs4775319	*RORA*	Expression pattern in lamina II of SC, a region for relaying somatosensory signals of touch, temperature and pain	Neuropathic pain	DNA/HNC	1,043	Reyes-Gibby et al./2018 [76]	1.59	1.36––1.87	1.02E-08
rs4804217	*PCP2*	Neuron-specific modulator of intracellular signaling via G proteins	Neuropathic pain	DNA/HNC	1,043	Reyes-Gibby et al./2018 [76]	0.58	0.48––0.69	2.95E-09

### Variants associated with mixed types of radiotherapy induced acute pain

Our literature search revealed only one study identified SNPs associated with post RT-pain in HNC without classification of the type of pain, we considered this phenotype as mixed types of pain. Liu et al. [77] study did not specify the grading scale used for toxicity identification; however, they divided patients into two groups of with or without toxicity of radiation at three, six and subsequent months. Age, gender, Body mass index (BMI), stage and ^131^I dose were used as covariates. 5 variants in 4 genes associated with RT-induced throat/neck pain post RT. [77]. (Table 5, Table 6).

**Table 5:** Summery of literature search of variants associated with other mixed types of RT-induced acute pain.

SNP	Gene	Pathway	Phenotype	Sample/cancer	Number of patients	Author/year	OR	95% CI	*P value*
rs1800629	*TNFα*	NF-κβ pathway, (Inflammation)	Neck pain/throat pain	DNA/Thyroid cancer	203	Liu et al./2018. [77]	10.3	2.7–39.1	0.001
rs11212570	*ATM*	DNA damage response (DDR)	Throat pain	DNA/Thyroid cancer	203	Liu et al./2018. [77]	4.3	1.4–13.1	0.01
rs230493	*NF-κβ*	Inflammation	Throat pain	DNA/Thyroid cancer	203	Liu et al./2018. [77]	0.2	0.1–1.0	<0.05
rs1800469	*TGF-β*	Cell growth, cell differentiation, vascular regeneration and apoptosis	Throat pain	DNA/Thyroid cancer	203	Liu et al./2018. [77]	5.543	1.1–28.8	0.04
rs2241716	*TGF-β*	Cell growth, cell differentiation, vascular regeneration and apoptosis	Throat pain	DNA/Thyroid cancer	203	Liu et al./2018. [77]	6.1	1.5–25.6	0.01

**Table 6:** Pathways and biological functions of genes associated with mixed types of radiotherapy induced acute pain.

Pathway	Genes	Number (n)	Percent (%)
Inflammatory/Immune system	*TNFα, NF-κβ*	2	50%
Cell death/Apoptosis	*TGF-β*	1	25%
DNA damage/DNA repair	*ATM*	1	25%

## Discussion

Acute pain is a significant toxicity during and after RT in HNC, resulting in an increase in disability and morbidity risks and a decrease in QOL of these patients. Understanding the potential pain mechanisms and the host genetic variability can help in pain prediction, prevention and in decision making for personalized pain management. To date, few studies explore the mechanisms of acute pain in HNC patients receiving RT. Moreover, very few variants have been identified associated with RT-induced acute pain in HNC. In this systematic review, we studied the different potential mechanisms for acute pain developed during RT in HNC cancer patients. SNPs to be identified associated with RT-induced acute pain in its different types (i.e., inflammatory/nociceptive, neuropathic, and mixed) in HNC studies were collected.

Our review found that inflammation, DNA damage/repair and cell death induce inflammatory pain following RT. Variants detected in genes involved in DNA damage response or DNA repair are associated with acute toxicities (i.e., mucositis and dermatitis) related to RT. Several studies evaluated the association of variants in DNA damage and DNA repair with RT-induced acute toxicities, 4 studies focused on variants in X-Ray Repair Cross Complementing 1 gene (*XRCC1*) as a DNA repair gene highly associated with RT toxicity [61], [63], [73], [74]. Our review identified other variants in DNA damage/repair genes (*ZNF24* [69], *NBN* [71], and *Ku70* [72]*)*. These results match our study of the potential mechanisms of inflammatory pain induced by RT in HNC [22]. Several studies identified SNPs in genes functioning in DNA damage and repair in other types of cancers such as; rs3218556 in *XRCC2* and rs13181 in *XPD* were associated with mucositis in non-small-cell lung cancer (NSCLC) [78], rs61915066 in *ATM*, rs11220184 in *CHEK1*, rs302877 and rs405684 in *RAD51C*, and rs60152947, rs10404465, rs1799786 in *ERCC2* which were associated with early adverse skin reaction (i.e., dermatitis) post-RT in breast cancer [79] and rs1233255 in *PMS1* gene was associated with RT-acute dermatitis in rectal cancer [80]. These variants could be explored and validated in HNC in future studies. Additionally, 7 studies identified variants in genes involved in inflammatory pathways and immune systems including; *TNF-α* [60], [77], *STING1* [57], *HLA-DQB1* [58], *TNFRSF1A* [64], *GHRL* [65], and *NF-κβ* [77], these variants were associated with RT-induced inflammatory pain and mixed throat pain. Studies showed that activation of inflammatory and immune cells after RT induces release of inflammatory cytokines and chemokines promoting more cell damage inducing inflammatory pain in addition to activation of nociceptors causing nociceptive pain [32], [33], [81], [82], [83]. Reyes-Gibby et al., studied the role of cytokines in pain activation and sensitization [53], [84]. Variants in genes regulating release of pro-inflammatory mediators such as cytokines and chemokines are shown here in our review. Other cell signaling pathways including cell cycle and *NF-kB* pathway (*CCND1*) [67], autophagy (*ATG16L2*, *ATG10*) [62], wnt/β-catenin (*APC*, *GSK3β*) [70] and angiogenesis (*EDN1*) [68] regulating genes were associated with RT related acute mucositis and dermatitis.

Few studies focused on neuropathic pain induced by RT, Reyes-Gibby et al. identified 4 SNPs in 4 genes (*SNX8*, *PCP2*, *RORA* and *KNG1*) correlated with neuropathy in HNC [76]. Although substance-P and other neurotransmitters exaggerate pain response [53], [85], no study identified any variant in substance-P or other neurotransmitters correlated with RT-induced pain. More studies are needed to identify the SNPs related to RT-induced neuropathy and neuropathic pain, especially variants in genes involved in neuronal plasticity and neuroinflammatory pathways.

Studies included in this review used either candidate gene approach or genome wide association analysis approach (GWAS), and interestingly through GWAS studies collected, we declared variants in genes involved in new pathways other than the common potential mechanisms, associated with RT-induced acute pain and toxicities in HNC. Sckack et al. conducted GWAS in HNC patients and identified candidate (rs1131769) in *DNAJC18* gene regulating a chaperon molecule protecting cellular proteins, significantly associated with OM after RT [57]. Yang et al., investigated GWAS of NPC patients and identified variant (rs117157809) in Tankyrase (*TNKS*) gene regulating telomere capping and maintenance of telomerase activity, is significantly associated with RT-induced OM [59]. Li et al., identified GWAS risk loci (rs1265081) in *CCHCR1* gene regulating cellular process as keratinocyte proliferation, differentiation, and epidermal growth factor receptor (*EGFR*) pathways, associated with RT-induced OM in HNC patients [58].

Although we did a comprehensive review to identify different potential mechanisms, including molecular pathways and genetic variants associated with RT-induced acute pain in HNC, the limitations in our review include that we did not explore SNPs associated with analgesics response (i.e., pharmacogenomics) or chronic pain. Our search focused on specific phenotypes in HNC population, although few studies were identified focused on HNC. Other variants identified in other cancer types with different phenotypes causing acute pain (e.g., pneumonitis, dysphagia, esophagitis, proctitis or bone pain) could be explored in detail in future studies. Furthermore, most of studies did not specify the type of pain detected, either inflammatory, nociceptive, or neuropathic, while they only mentioned post-RT pain, mucositis, or dermatitis. We involved variants associated with unspecified post RT-pain under the group of other types/mixed pain. These collected studies are just association studies; however, mechanistic studies must be done to test the mechanistic effect of these variants on the phenotype development and alteration of the different pathways associated. The identified candidates need to be studied further for validation of results.

Our future directions include validating these variants and developing predictive algorithms for pain prediction and management using genetic candidates identified in our HNC cohort.

## Conclusion

This comprehensive literature review aimed to identify the genetic variants association with RT-induced acute pain phenotypes including; mucositis, dermatitis (Inflammatory pain), neuropathic, nociceptive pain and mixed pain in HNC population. Our review revealed that pain is a complex symptom during and following RT in HNC patients due to its multifactorial origin and diverse phenotype expression. DNA damage/repair, inflammatory pathways, apoptosis, and neuropathy pathways are the most common pathways behind the development of RT-related acute pain. There is a need for a comprehensive understanding of the genetic profile risk association with acute pain severity, additionally, the clinical findings and treatment plans for HNC patients may help inform development of standardized algorithms for personalized management of acute RT-induced pain to maintain pain relief during RT and improve patients’ outcome.

## Funding statement

This work was funded by Paul Calabresi K12 Scholars Program, 5K12CA088084, Pain-directed Pragmatic Automation of Informatics-based Novel Frameworks for Reducing Radiotherapy-associated Effects (PAINFREE) offered to AM. VS is funded by The University of Texas Graduate School of Biomedical Sciences Graduate Research Assistantship. JR is funded by NIDCR 3R01DE028290-02S1.

## Contribution Author(s)

Study concepts: Vivian Salama, Clifton D. Fuller, Sanjay Shete, Amy C. Moreno.

Study design: Vivian Salama.

Data acquisition: Vivian Salama, Yimin Geng, Jillian Rigert.

Quality control of data and algorithms: Vivian Salama, Jillian Rigert, Clifton D. Fuller, Amy C. Moreno.

Data analysis and interpretation: Vivian Salama.

Statistical analysis: Vivian Salama.

Manuscript preparation: Vivian Salama, Yimin Geng.

Manuscript editing: Vivian Salama, Jillian Rigert, Clifton D. Fuller, Amy C. Moreno.

Manuscript review: Vivian Salama, Jillian Rigert, Clifton D. Fuller, Sanjay Shete, Amy C.

## Declaration of Competing Interest

The authors declare the following financial interests/personal relationships which may be considered as potential competing interests: CF has received direct industry grant support, speaking honoraria, and travel funding from Elekta AB. The remaining authors declare that they have no known competing commercial, financial interests or personal relationships that could be constructed as potential conflict of interest.
